# Spatial and developmental synthesis of endogenous sesquiterpene lactones supports function in growth regulation of sunflower

**DOI:** 10.1007/s00425-020-03409-y

**Published:** 2020-06-05

**Authors:** Otmar Spring, Katharina Schmauder, Nathalie D. Lackus, Jasmin Schreiner, Carolin Meier, Jan Wellhausen, Lisa V. Smith, Maximilian Frey

**Affiliations:** Institute of Biology, University of Hohenheim, Garbenstraße 30, 70593 Stuttgart, Germany

**Keywords:** Gene expression, Growth inhibition, *Helianthus*, Light induction, Sesquiterpene lactone biosynthesis

## Abstract

**Main conclusion:**

Tissue-specific occurrence and formation of endogenous sesquiterpene lactones has been assessed and suggests physiological function as antagonists of auxin-induced plant growth in sunflower.

**Abstract:**

Sunflower, *Helianthus annuus*, accumulate high concentrations of bioactive sesquiterpene lactones (STL) in glandular trichomes, but in addition, structurally different STL occur in only trace amounts in the inner tissues. The spatial and temporal production of these endogenous STL during early phases of plant development is widely unknown and their physiological function as putative natural growth regulators is yet speculative. By means of HPLC and MS analysis it was shown that costunolide, dehydrocostuslactone, 8-epixanthatin and tomentosin are already present in dry seeds and can be extracted in low amounts from cotyledons, hypocotyls and roots of seedlings during the first days after germination. Semi-quantitative and RT-qPCR experiments with genes of the key enzymes of two independent routes of the endogenous STL biosynthesis confirmed the early and individual expression in these organs and revealed a gradual down regulation during the first 72–96 h after germination. Light irradiation of the plants led to a fast, but transient increase of STL in parts of the hypocotyl which correlated with growth retardation of the stem. One-sided external application of costunolide on hypocotyls conferred reduced growth of the treated side, thus resulting in the curving of the stem towards the side of the application. This indicates the inhibiting effects of STL on plant growth. The putative function of endogenous STL in sunflower as antagonists of auxin in growth processes is discussed.

**Electronic supplementary material:**

The online version of this article (10.1007/s00425-020-03409-y) contains supplementary material, which is available to authorized users.

## Introduction

For a long time, sesquiterpene lactones (STL) of sunflower *Helianthus annuus* L. were thought to be only produced and stored in trichomes of leaves or flowers (Spring et al. 1985, 1987, 1989a) with the function to protect the plant against predators (Spring et al. 1989b; Mullin et al. 1991). However, in the late 1990s a first report on the presence of 8-epixanthatin (8-EPI), a STL of the xanthanolide type, in extracts of sunflower hypocotyls was published (Yokotani-Tomita et al. 1997). The fast accumulation of 8-epixanthatin after blue light irradiation and the inhibition of elongation growth upon unilateral external application on hypocotyls suggested a functional role of the compound in growth regulation (Yokotani-Tomita et al. 1999). More recently, additional STL such as dehydrocostuslactone (DCL) (Joel et al. 2011), costunolide (COS) and tomentosin (TOM) (Raupp and Spring 2013) were found in roots and in root exudates, from where they diffuse into the soil. There, the STL act as allelopathic compounds which stimulate the germination of broomrape (*Orobanche cumana* Wallr.) seeds and act as a signal for the chemotropic orientation of the parasite’s germtube (Krupp 2020). The much lower concentrations of these endogenous STL (nM to low µM) compared to the externally deposited STL in trichomes (mM concentrations) makes them less suitable as toxic antifeedants, but rather suggests an internal physiological function of these metabolites with an independently regulated pathway (Padilla-Gonzalez et al. 2016).

**Fig. 1:**
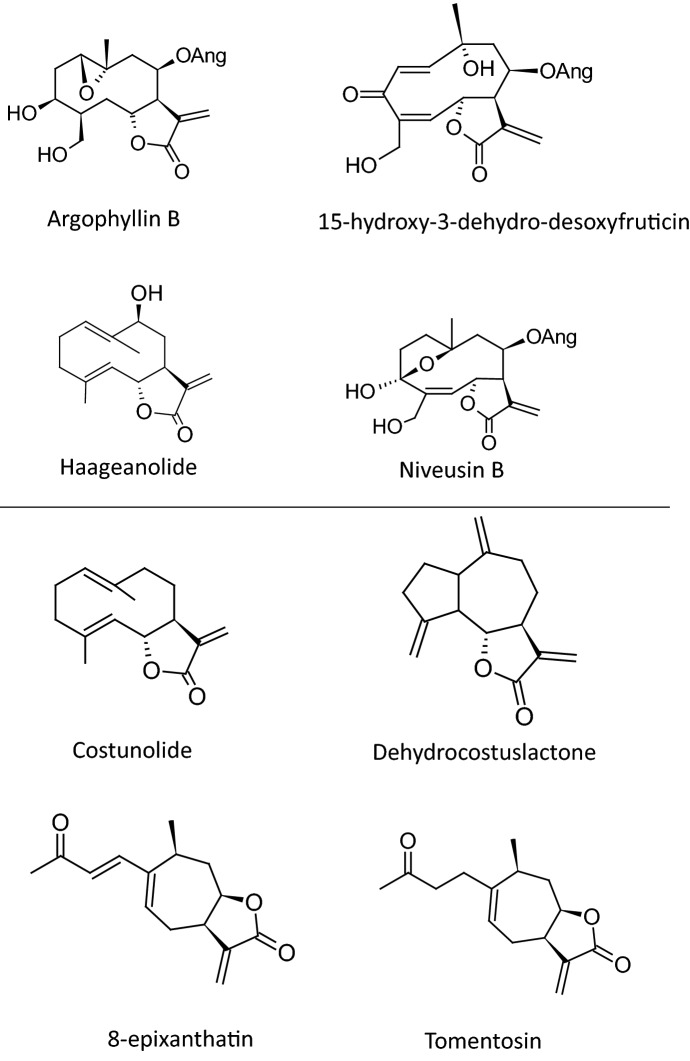
Examples of trichome-based (above line) and endogenous (below line) sunflower sesquiterpene lactones

**Fig. 2:**
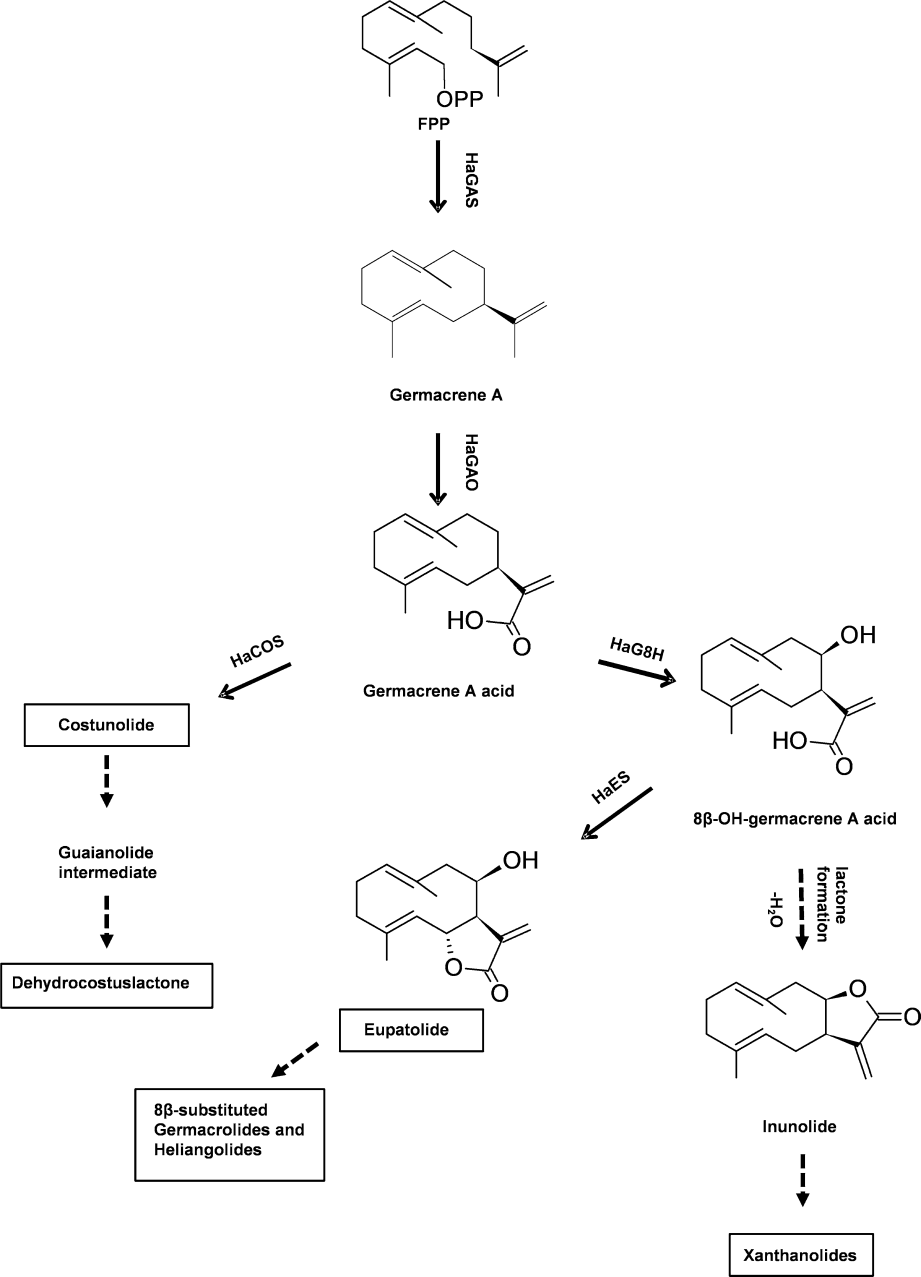
Proposed biosynthetic pathway of costunolide, dehydrocostuslactone, xanthanolides and 8β-substituted germacrolides and heliangolides in sunflower. Solid arrows mark enzymatically proven steps in sunflower. FPP, farnesylpyrophosphate synthase; HaGAS, *Helianthus annuus* germacrene A synthase; HaGAO, *Helianthus annuus* germacrene A oxidase; HaG8H, *Helianthus annuus* 8β-hydroxylase, HaCOS, *Helianthus annuus* costunolide synthase

This assumption is supported by the structural differences found between the internal and external STL (Fig. 1). While the trichomes of sunflower harbor heliangolides and germacranolides usually with ester side-chains at C-8 (Spring et al. 1986; Prasifka et al. 2015), the four identified endogenous STL lack side chains and consist of three structurally different skeletal categories: two xanthanolides (8-epixanthatin, tomentosin), a guaianolide (dehydrocostuslactone) and a germacranolide (costunolide). This seems to require considerable differences in the enzymatic patterns necessary for the biosynthesis of these compounds. However, recent studies in STL biosynthesis of sunflower revealed that all three of these skeletal types are based on the pathway of germacrene A acid (Fig. 2). This intermediate is formed from farnesyl pyrophosphate (FPP) in two enzymatic steps by germacrene A synthase (HaGAS) (Göpfert et al. 2009) and germacrene A oxidase (HaGAO), a cytochrome P450 enzyme which introduces a carboxylic group at C-12 (Nguyen et al. 2010). The decisive step for the skeletal type is the subsequent hydroxylation through additional P450 enzymes which stereospecifically either introduce an α-hydroxyl at position C-6 through the costunolide synthase HaCOS (Frey et al. 2020) or a β-hydroxyl at C-8 of the germacrene ring through the germacrene A acid 8β-hydroxylase HaG8H (Ikezawa et al. 2011), respectively. The subsequent spontaneous internal esterification with the C-12 carboxyl in the first case leads to the trans-7,6-lactone costunolide, from which the guaianolide skeleton of dehydrocostuslactone can derive in further steps (Álvarez-Calero et al. 2018; Liu et al. 2018). In the second case, the cis-7,8-lactone inunolide is formed, which is an intermediate in the biosynthesis to the two xanthanolides 8-epixanthatin and tomentosin. Contrary to this pathway for the endogenous STL, in sunflower trichomes HaCOS is functionally replaced by the eupatolide synthase HaES (Frey et al. 2018), which in co-expression with HaG8H forms trans-7,6-lactones with a C-8 β-hydroxyl group, from which the typical STL with angelic ester side chain are formed.

While the formation and storage of STL in sunflower trichomes had been studied intensively (Göpfert et al. 2005; Amrehn et al. 2014, 2016; Aschenbrenner et al. 2015), the knowledge on the metabolism and physiological function of the endogenous STL in different organs of the plant so far remained fragmentary. This was partially due to the extremely low concentrations of the STL in plant tissues of specific physiological stages, which requires highly advanced chromatographic techniques for their detection. In addition, questions on gene expression of enzymes necessary for the biosynthesis of the endogenous STL could not be addressed until the involved genes had been identified very recently (Frey et al. 2018, 2020). The present study now aimed to shed light into the biosynthesis and occurrence of STL in the inner tissues of sunflower. This was investigated by gene expression analysis of key enzymes in different plant organs and developmental stages of sunflower seedlings. In addition, STL metabolite profiles from root, stem and cotyledon extracts were analyzed via HPLC–UV or HPLC–MS/MS measurements. The four STL were quantitatively compared in the course of germination, during early plant development and under the influence of light irradiation. The correlation between STL occurrence, gene expression and plant growth processes was assessed and is discussed with respect to the putative physiological function of these compounds in plant growth processes.

## Materials and methods

### Plant material and cultivation

The sunflower *H. annuus* L. line HA300 and the cultivar Giganteus (Dürr Samen Schwenk e.K. Reutlingen, Germany) were used for this study. Seeds were germinated on moistened paper tissue in plastic boxes at 22 °C for up to 48 h in darkness. Thereafter, the seed hulls were completely removed; the seedlings were thoroughly washed for 1 h in tap water and then transferred to plastic boxes for hydroponic cultivation in tap water (40 seedlings per 0.5 l box). Plants were grown in a climate chamber at 22 °C, 80% humidity and a light regime of 14 h white light (100 µM/m^2^ s) followed by 10 h darkness per day.

### STL extraction and analysis

For the extraction of metabolites, 50–100 seedlings per sample were harvested, dissected in cotyledons (frontal 65% only, to avoid contamination with primordia of true leaves), hypocotyls (5 mm below the cotyledons) and roots (frontal 2–3 cm). The pooled organ samples were weighed, placed in 2 ml reaction tubes, shock frozen in liquid nitrogen and then stored at − 70 °C until extraction. To avoid the possible influence of circadian rhythm or a different length of light irradiation (Spring et al. 1986; Yokotani-Tomita et al. 1999), the harvest started always 6 h after the beginning of the light phase and the preparation was performed under green light.

For extraction, the frozen samples were homogenized in 2 ml reaction vials with steel balls (2 mm, Sarstedt, Nürnbrecht, Germany) in a mixer will (MM400 Retsch, Haan, Germany), spiked with ethyl acetate and stirred for 5 min. The solvent phase was separated after centrifugation (8000*g*, 3 min) and the extraction with ethyl acetate was repeated twice. The pooled solvent extracts were dried under reduced pressure in a vacuum concentrator, dissolved in methanol and diluted to 50% with water. Insoluble particles were sedimented by centrifugation (8000*g*, 15 min, and 4 °C) before the cleared samples were used for HPLC analysis.

In the case of seeds (dry or moistened for 24 h), the extraction had to be modified due to the high content of the oil. The dehulled seeds were chopped with a razor blade and then homogenized with steel balls in the mixer mill in the presence of methanol (500 µl per 5 seeds). After centrifugation (8000*g*, 3 min, 4 °C), the methanolic phase was separated and the residue was treated twice again in the same way. The three methanol phases were pooled and evaporated in a vacuum concentrator. The resinous residue was dissolved in deionized water in an ultrasonic bath and then diluted 1:1 (v/v) with acetonitrile. The dull samples were stored over night at 4 °C and cleared through centrifugation immediately before HPLC analysis.

The efficacy of STL extraction from the different plant’s organs was assessed by spiking homogenized material, which had been extracted before 3 times with methanol, with costunolide and dehydrocostuslactone in defined amounts (Supplementary Table S1).

### HPLC–UV and HPLC–MS analysis

HPLC–UV analysis of samples was performed in a two-step mode using a Shimadzu SIL-20A unit with diode array detector SPD-M10AVP for the first separation on a C18 Kromasil column (5 µm particle size, 250 × 4 mm; MZ Analysetechnik, Mainz, Germany) with a methanol–water gradient (55–65% in 20 min, 65–100% in 15 min; flow 1 ml/min). The retention times of the 4 STL were determined by means of reference compounds (costunolide from Absource Diagnostics GmbH; München, Germany; dehydrocostuslactone from Cfm Oskar Tropitzsch GmbH, Marktredwitz, Germany; tomentosin and 8-epixanthatin were not commercially available and had to be extracted and purified from *Xanthium strumarium* leaves in our lab). Peak detection was made at 210 nm for costunolide, dehydrocostuslactone and tomentosin, and at 278 nm for 8-epixanthatin. Quantification was performed by measuring the peak area and calculating the quantity in comparison to calibration curves of the reference compounds.

As in some cases, the target peaks were very small or partly overlapped by other compounds in the tissue extract, manual peak fractionation was conducted in the first separation step. The fractions were dried in a vacuum concentrator, resolved in 30% (tomentosin, 8-epixanthatin) or 50% (costunolide, dehydrocostuslactone) acetonitrile and analyzed in a second run on a Dionex P580 HPLC with UVD 340 S-Detector (Dionex, Sunyvale, CA, USA) equipped with a RP 18 Gromsil column (120 ODS ST, 3 µm, 150 × 4.6 mm; Alltech GROM, Worms, Germany). Gradients of acetonitrile–water (30–50% in 20 min for tomentosin and 8-epixanthatin samples; 50–70% in 15 min for costunolide and dehydrocostuslactone samples) were used for this second separation and quantification of target peaks was performed again by means of calibration curves with reference compounds.

In cases where the amount of extracted STL was too low for reliable quantification in UV diagrams of normal HPLC, samples were analyzed by HPLC–MS/MS on an Agilent 1290 UHPLC system (Agilent, Waldbronn, Germany) coupled to a *Q Exactive Plus* (Thermo Scientific, Schwerte, Germany) high-resolution tandem mass spectrometer. Chromatographic separation of plant extracts was performed on an Acquity HSS T3 column (1.8 μm, 50 mm × 2.1 mm; Waters, Dublin, Ireland) using an acetonitrile–water (0.1% formic acid) gradient (10–80% in 15 min) as a solvent. Mass spectrometry detection was performed in positive ionization mode using a heater temperature of 600 °C, spray voltage of + 5.5 kV, and nitrogen as the collision gas. Total Ion Current (TIC) chromatograms were obtained over the range of 140–1200 *m/z*.

Peak identity was determined by comparing the high-resolution mass of molecule peak (deviance range 5 ppm), fragmentation patterns, and HPLC retention times in comparison to the authentic reference compounds. For quantification, the MS^+^ peak intensity of the mother ion was used: 233.1535 for costunolide; 231.1379 for dehydrocostuslactone, 249.1484 for tomentosin, and 247.1327 for 8-epixanthatin. Due to the instability of tomentosin, the first fragment peak ([M–H_2_O]^+^ with 231.1379) was higher than the molecule peak and therefore was also used for quantification. For the calculation of STL concentration, the sample fresh weight, extraction efficacy and the relative injection volume was considered. The intensity of the MS^+^ peak ([M–H_2_O]^+^ peak for tomentosin) from calibration runs with defined amounts of the references was used to calculate the absolute concentrations in the plant tissues.

### RNA preparation

The plant material, harvested and frozen in nitrogen as described above, was used for total RNA extraction with the Spectrum™ Plant Total RNA Kit (Sigma-Aldrich, Steinheim, Germany). The samples were homogenized using two steel balls (2 mm, Sarstedt) in a mixer will (MM400 Retsch) for 1 min at 30 Hz. After cooling the sample, this step was repeated 3 times. After cell disruption, 500 μl of lysis buffer was added. All subsequent steps were carried out as described in the manufacturer’s manual.

RNA quantity and purity was verified photometrically (Bio Photometer; Eppendorf, Hamburg, Germany). Before cDNA synthesis, the RNA concentration of all samples was adjusted with RNAse-free H_2_O to 10 ng/µl. The cDNA was synthesized in a PeqSTAR-Cycler (PeqLab 96 universal gradient, VWR International, Erlangen, Germany) using the RevertAid First Strand cDNA Synthesis Kits (Fermentas, St. Leon-Rot, Germany) with Oligo(dT) primer as described in the manual.

The purity of the cDNA was tested by PCR with HexaUbiQ primers (Grasse 2015) which deliver a 244 bp fragment for sunflower with cDNA in contrast to a 1200–1500 bp product with gDNA. In the case of gDNA contamination, the RNA of the sample was treated with PerfeCTa-DNAse I (Quanta Bioscience™, Gaithersburg, MD, USA) as described in the manual and the cDNA synthesis was repeated.

### Expression studies

The cDNA samples from different plant organs were used for semi-quantitative PCR with the following primer pairs specific for genes of key enzymes of the STL biosynthesis in sunflower: *farnesylpyrophosphate synthase FPS,* 5′-ACT GCT TGT ACG GCT TTG CTT G-3′, 5′-TTT CTT GCA TCT GCC CTT GGT TG-3′ (Göpfert et al. 2009); germacrene A synthase *HaGAS*, 5′-CCC TCC TGT TTG TTG GTT CTT CAC CC-3′, 5′-ACG AGG GGT GTCTCA AGC CA-3′ (this paper); germacrene A oxidase *HaGAO*, 5′-GAC CGA GAG CTT TGG AGC CAC G-3′, 5′-CGG CAC TCG GTC TAG CAA ACG T-3′(this paper); germacrene A acid 8-hydroxylase *HaGA8H*, 5′-CGA AAC CTT AGA AAC TTG GCT-3′, 5′-AAT AAA CTG TCG GTC TTC GCT AAC-3′ (this paper). The constitutively expressed ubiquitin was used as a reference gene. A partial sequence of 420 bp was amplified with the primers 5′-CAA AAC CCT AAC CGG AAA GA-3′, 5′-ACG AAG ACG GAG GAC GAG-3′ (Göpfert et al. 2009). The cDNA of different samples was adjusted according to equal amplification intensity for the ubiquitin transcript. The PCR reactions were run in a peq-STAR Thermocycler (Peqlab, Erlangen, Germany) with Fermentas Taq DNA Polymerase under following conditions: 3 min initial denaturation (94 °C), followed by 36 cycles of 40 s annealing (59 °C), 1 min elongation (72 °C) and 3 min final elongation (72 °C).

The evaluation of the amplification products was performed by capillary electrophoresis on a MCE220 MultiNA (Shimadzu, Kyoto, Japan) with control and MultiNA Viewer software and microchips with DNA 500 and 1000 kit. Two to three biological repetitions each and three technical repetitions were carried out for each detected gene.

In a subsequent series of experiments, the quantitative expression on STL genes was analyzed using qPCR with a CFX96TM C1000 TouchTM- Thermal Cyclers (BioRad, Feldkirchen, Deutschland) in combination with software CFXTM-Manager (version 3.0, BioRad). RNA extraction and cDNA synthesis were performed as described above. Primers suitable for qPCR were designed with PrimerSelect (Version 10.0.1 (3), DNASTAR^®^ Inc., Madison, WI, USA). The used primer sequences for *Helianthus annuus germacrene A oxidase (HaGAO), Helianthus annuus 8β*-*hydroxylase (HaG8H), Helianthus annuus costunolide synthase (HaCOS), α*-*tubulin, actin, elongation factor 1α,* the GeneBank accession numbers, and the CYP numbers are shown in Supplementary Table S2. The primer specificity was checked according to the melting curves and the identity of the amplification products was verified by sequencing (Macrogene, Amsterdam, The Netherlands). The primer efficacy was determined as linear by testing dilutions of cDNA in the range of 1:2 to 1:512. For statistical analysis, the SE was calculated from three biological and three technical replicates.

The qPCR experiment was carried out in 12.5 µl sample volume (6.25 µl SensiFAST SYBR NoRox Mix (BioLine, Luckenwalde, Deutschland), 4.25 µl ddH_2_O, 0.5 µl of each primer and 1 µl template cDNA) with the following program: 2 min initial denaturation (95 °C), followed by 39 cycles of 15 s denaturation (95 °C), 15 s annealing/elongation (60 °C) and finished with a melting curve in which the temperature was raised from 65 to 95 °C in steps of 0.5 °C.

The calculation of the relative normalized gene expression (RNE) was performed with the program CFXTM-Manager (Version 3.0, BioRad). All three reference genes and the individual primer efficacy were included in the calculation. Significance of statistical differences between three biological and three technical repetitions was tested by one-sided ANOVA and *t* test using qbase^+^ (version 3.0, Biogazelle, Gent, Belgium).

### Irradiation experiments

Sunflower seedlings germinated and cultivated as described above in hydroponic culture for 5 days in darkness were used to test the fluctuation of the STL content under the light influence. Half of the plants were kept in a day/night regime with 14 h per day white light (100 µmol/m^2^ s; 380–750 nm) irradiation, whereas the second half was continuously kept in darkness as control. Hypocotyls of 50 seedlings per sample were harvested at different time points after the beginning of irradiation: *T*_0_ (start of irradiation); *T*_2_, *T*_24_ and *T*_48_. Segments of 0.5 cm were cut out from the upper hypocotyl (ca. 3 mm below the hook) and the lower hypocotyl (ca. 5 mm above the root), extracted and analyzed by HPLC as described above in the two-step manner to eliminate peak overlapping. The experiment was performed twice and the results showed similar trends. In addition, growth parameters (length of hypocotyl, bending of the hook and unfolding of cotyledons) were assessed (Supplementary Fig. S1 and Fig. S2).

In a second experiment, seedlings were kept under white light (14 h per day) until the cotyledons had been unfolded (5–6 days after germination). Afterwards, the plants were kept for 24 h in darkness to lower the internal level of STL. Then, under green safety light (560 nm LED), the plants were decapitated ca. 2.5 cm below the cotyledons and placed individually in 0.5 ml reaction vials filled with sucrose (0.2 M) solution (to avoid osmotic reaction). The vials were placed in a humid plastic container (100% rel. humidity to prevent transpiration) and plants were irradiated from the top with blue light (457 nm LED, 117 µM/m^2^ s^1^) for up to 4 h. Plants kept in darkness served as control. After irradiation, two segments (upper, lower part) of 1 cm in length were cut from the hypocotyl under green safety light, immediately shock frozen in liquid nitrogen and then stored at − 20 °C until extraction. Per sample (*T*_0_, *T*_2_ and *T*_4_ light and dark), 15 plants were used and the experiment was performed five times. The extraction was performed as described above and the dried residue was dissolved in 25 µl acetonitrile for subsequent analysis in HPLC–MS (4.3). Sample volumes of up to 7 µl were injected. Peak identification and quantification were performed as described before.

### Unilateral applications of costunolide

Sunflower seedlings, cultivated in the climate chamber for ca. 7 days (14 h light, 10 h dark per day) were selected for equal growth and planted individually in 15 ml plastic tubes. Before starting the experiment, the plants were kept in darkness for 2 h. Costunolide (500 µM final concentration) and dimethylsulfoxide (DMSO, 10% final concentration) were mixed with vaseline and applied unilateral on the left or right flank of the hypocotyl of plants with a spatula under safety green light. Identical applications of pure vaseline and vaseline with 10% DMSO served as controls. For the experiment, the plants were kept in darkness for 200 min and photographed every 20 min in green light to monitor the vertical position of the hypocotyl (determined as a straight line between bottom and apex). Deviation angles from the starting position (*T*_0_) of at least 8 plants per sample were determined as illustrated in Supplementary Fig. S3.

### Statistics

If not indicated differently, the experiments were performed with at least three biological replications, from which mean values and SE were calculated. Statistical significance was compared using ANOVA and Fisher LSD tests (*P *= 0.05 and *P *= 0.10) with the program InfoStat (Infostat Group 2014, Universidad Nacional de Córdoba, Argentina).

## Results

### Spatial and developmental occurrence of endogenous STL

**Fig. 3:**
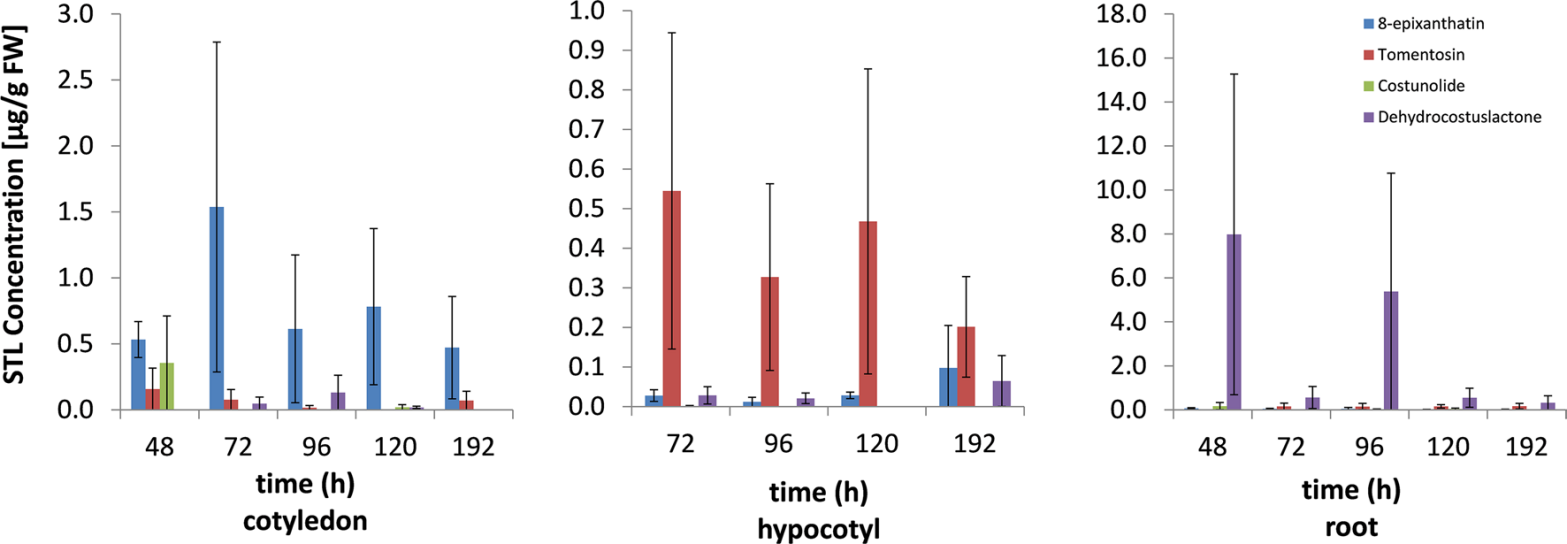
Temporal and spatial distribution of sesquiterpene lactones in cotyledons, hypocotyls and roots of sunflower seedlings between 48 and 192 h after imbibition. Quantification was performed by HPLC–UV using standard curves of reference compounds and the relative concentration of STL was corrected applying tissue- and compound-specific extraction efficiency. Mean values ± SE (*n *= 3)

Sunflower seedlings, germinated in darkness for 48 h and then raised up to 192 h in hydroponic culture with a 14 h light period per day were analyzed for the presence of STL in different plant organs. Extracts of cotyledons, hypocotyls (first samples available after 72 h) and roots in HPLC analysis showed peaks of the four known internal STL costunolide, dehydrocostuslactone, 8-epixanthatin and tomentosin in variable amounts (Fig. 3). While 8-epixanthatin dominated in cotyledons, tomentosin concentrations were highest in hypocotyls and dehydrocostuslactone reached highest amounts in roots. Costunolide, though detected in all three organs as well, was mostly present in very small amounts. The concentrations of 8-epixanthatin in cotyledons and tomentosin in hypocotyls showed a maximum in the 72 h samples and decreased over the following 5 days of cultivation. For dehydrocostuslactone in roots, this trend was less clear due to the inexplicable high variance of the results in two of the samples.

As cotyledon samples 48 h after imbibition showed detectable amounts of STL, the experiment was extended to the period before and to samples of non-germinated seeds. Again, the four compounds were detected in the extracts quantified by HPLC–UV and verified by HPLC–MS measurements. In non-germinated seeds, a total of 1.5 µg STL/g FW was found with tomentosin (1.0 µg/g FW) and costunolide (0.37 µg/g FW) being dominant. After 24 h of germination, the total concentration of the four compounds had dropped to 0.6 µg STL/g FW. While tomentosin had dropped by almost 90%, costunolide remained nearly unchanged (0.32 µg/g FW) and 8-epixanthatin raised from 0.04 to 0.13 µg/g FW. Dehydrocostuslactone was present in low concentrations (0.01 to 0.02 µg/g FW) throughout this period.

### Expression of genes involved in STL biosynthesis

**Fig. 4:**
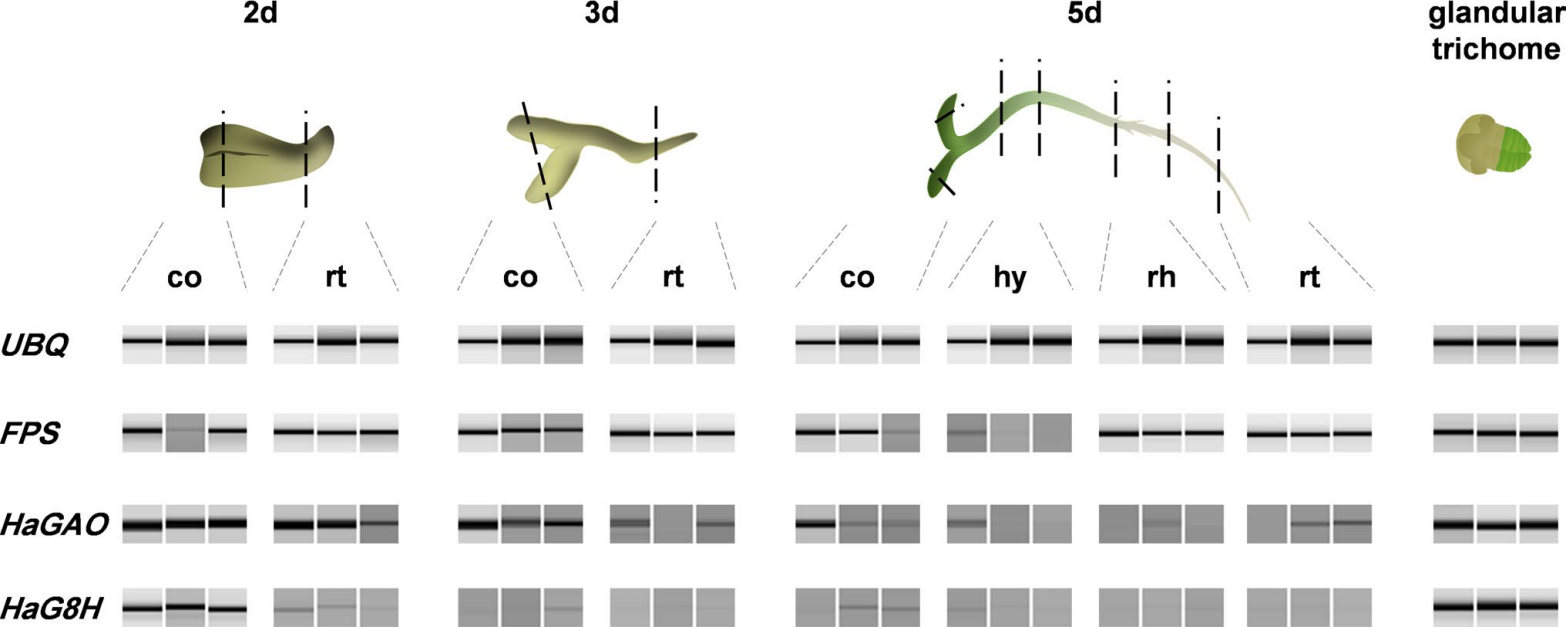
Semi-quantitative gene expression in organs of sunflower seedlings between two (2d), three (3d) and five days (5d) after imbibition. *Ubiquitin* expression was used as house-keeping gene. The data (generated by capillary electrophoresis) represent the relative expression of *FPS* (*farnesyl*-*pyrophosphate*-*synthase*), *HaGAO* (*Helianthus annuus germacrene A oxidase*) and *HaG8H* (*Helianthus annuus germacrene A acid 8β*-*hydroxylase*) of three independent experiments. Capitate glandular trichomes of sunflower were used as a positive control for biosynthetic activity of genes involved in trichome-based sesquiterpene lactone biosynthesis. *co* cotyledon, *hy* hypocotyl, *rh* root hair zone, *rt* root tip

To test the correlation of gene expression of the STL pathway with the spatial and developmental occurrence of STL, a semi-quantitative analysis of expressed genes was performed in samples from seedlings of 48–120 h past imbibition of seeds. Farnesyl-pyrophosphate-synthase (*FPS*), the initial step to sesquiterpene synthesis, was expressed almost homogeneously in all organs and over the whole time frame (except for older hypocotyl samples). The housekeeping gene *ubiquitin* was used as a reference (Fig. 4). The expression of the sunflower germacrene A oxidase *HaGAO* was strong in young cotyledons and roots, but gradually dropped in older seedlings. *HaG8H,* the sunflower germacrene A acid 8β-hydroxylase, showed strong expression in young cotyledons but was much weaker in roots and older stages of cotyledons.

**Fig. 5:**
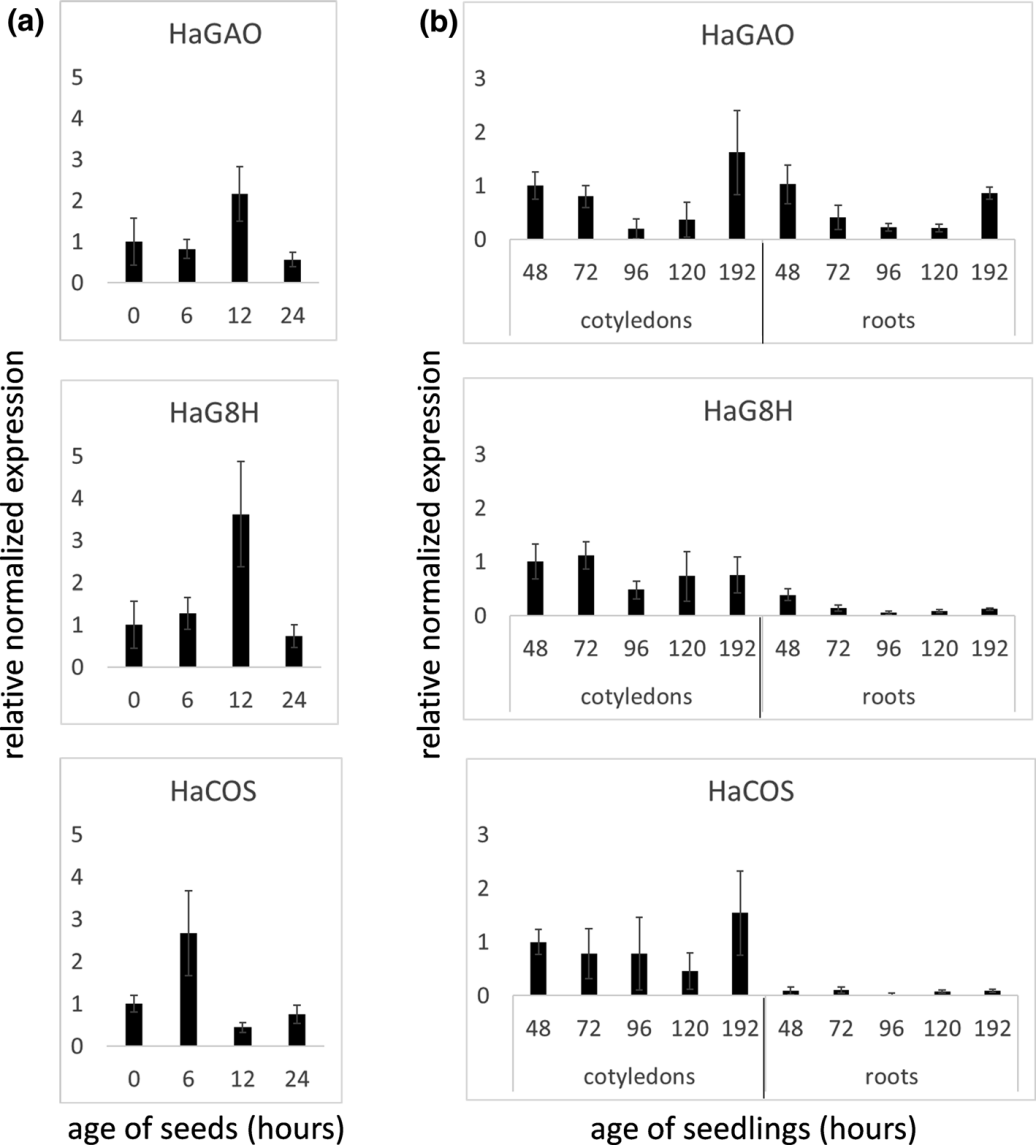
Quantitative expression of genes involved in sesquiterpene lactone biosynthesis of sunflower in **a** seeds and **b** seedlings at different times after imbibition. Expression values are normalized to three house-keeping genes (*α*-*tubulin, elongation factor 1α,* and *ubiquitin*) and are given relative the youngest stage of seeds (ungerminated seeds at 0 h) or the youngest cotyledon stage (48 h), respectively. For every biological replicate, three technical replicates were analyzed. Mean values ± SE (*n* = 3). *HaGAO*, *Helianthus annuus germacrene A oxidase*; *HaG8H*, *Helianthus annuus germacrene A acid 8β*-*hydroxylase*; *HaCOS*, *Helianthus annuus costunolide synthase*

Quantitative expression studies using three house-keeping genes (*α*-*tubulin, elongation factor 1α,* and *ubiquitin*) showed, that the key enzymes *HaGAO*, *HaG8H* (essential for the synthesis of tomentosin and 8-epixanthatin), and *HaCOS* (necessary for costunolide and dehydrocostuslactone) were already highly expressed in seeds and early germination stages (Fig. 5). Compared to non-germinated seeds (Fig. 5a; 0 h), the expression of all three genes raised by a factor of 2–3.5 within the first 6–12 h after imbibition and then dropped again. In cotyledons and roots of seedlings, the expression decreased from 48 to 96 h and then began to increase again towards the stage, when the hypocotyl growth faded and primary leaves started to develop (Fig. 5b; 8 days = 192 h). While *HaGAO* expression was similar in cotyledons and roots, *HaG8H* and *HaCOS* were substantially less expressed in roots.

### Light influence on STL in hypocotyls

Etiolated sunflower seedlings at the age of 5 days had hypocotyls of 1.78 ± 0.58 cm in length (average of *n* = 100 plants) at the beginning of the experiment. They showed a nearly linear growth of ca. 1.02 cm per day within the next 48 h when further kept in darkness (Supplementary Fig. S1). Their yellowish cotyledons stayed folded and pointed downwards when the plants were further kept in darkness. Contrary, hypocotyls grew only by 0.27 cm per day when the etiolated seedlings were cultivated under a light regime of 14 h white light/10 h darkness. In addition, the hypocotyl hook became straight, the cotyledons unfolded and the photosynthetically active tissues turned green (Supplementary Fig. S2).

Hypocotyl extracts of two independent experiments were analyzed by HPLC–UV. Both series showed a fast increase of the xanthanolides 8-epixanthatin and tomentosin in the hypocotyl sections at 2 h to 24 h past the first illumination, although with very high differences in the absolute concentrations. The concentration (mean of the two series) rose from 49 ng/g FW (SE 9.8) at *T*_0_ to 1272 ng/g FW (SE 690) at *T*_24_ for 8-epixanthatin and from trace amounts at *T*_0_ to 82 ng/FW (SE 80) at *T*_24_ for tomentosin. These values subsequently dropped to 68 (SE 21) and 37 ng/g FW (SE 36), respectively, in the *T*_48_ samples. Control plants, continuously kept in darkness, did not show increasing amounts of the compounds over the time frame tested and the *T*_48_ samples contained only traces of both STL. Costunolide and dehydrocostuslactone were only found in trace amounts in dark as well as irradiated samples and could not be quantified by HPLC–UV. However, their presence in the hypocotyl at *T*_0_ and *T*_24_ could be verified by HPLC–MS (data not shown).

**Fig. 6:**
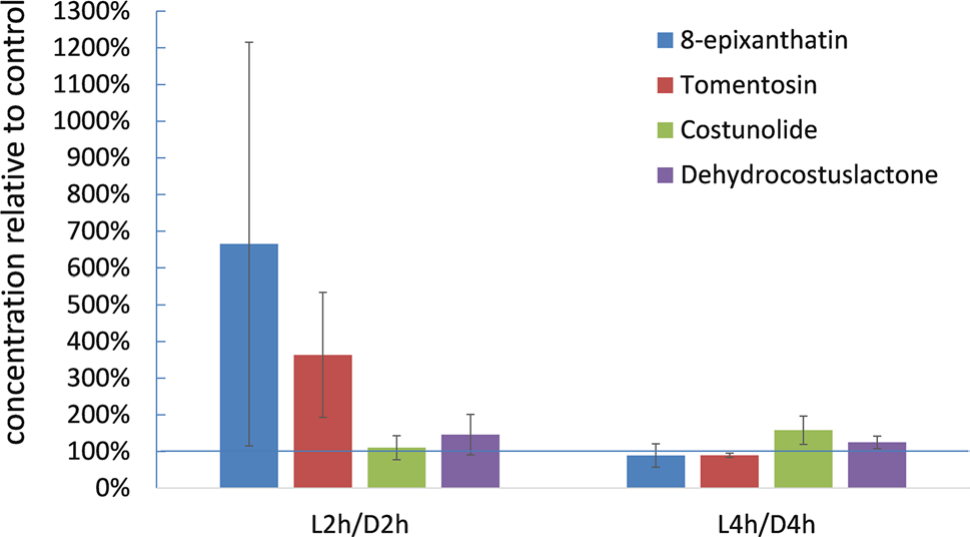
Relative concentration of sesquiterpene lactones in the hypocotyl of sunflower seedlings after 2 h (L2h) and 4 h (L4h) of illumination in comparison to dark controls (D2h, D4h; defined as 100%; blue horizontal line). Mean values ± SE (*n* = 3) are displayed. *COS* costunolide, *DCL* dehydrocostuslactone, *8-EPI* 8-epixanthatin, *TOM* tomentosin

In a second, but the modified approach, seedlings were raised for 5–6 days in white light (14 h per day) and then etiolated for 24 h before cutting the hypocotyl approximately 2.5 cm below the cotyledons. Extracts of the hypocotyl were made 2 h (*T*_2_) and 4 h (*T*_4_) after irradiation of the cotyledons from the top with blue light (457 nm LED, 117 µmol/m^2^ s^1^). HPLC–MS/MS analysis showed the presence of small amounts of all four endogenous STL in dark controls of both plant parts in concentrations of around 26, 7, 166 and 14 ng/g FW for 8-epixanthatin, tomentosin, costunolide and dehydrocostuslactone, respectively. After 2 h of irradiation, the relative concentration of 8-epixanthatin and tomentosin in the hypocotyl raised to 670% and 325%, respectively, of the dark controls, whereas dehydrocostuslactone and costunolide were almost unaffected. After 4 h of illumination, the concentration of the two xanthanolides had readjusted to values of the dark controls (Fig. 6). The effect of 2 h irradiation on the concentration of tomentosin was significant in the LSD test (*P *= 0.05; Supplementary Table S3), whereas the strong increase in 8-epixanthatin failed significance due to the high variation of the measured data. Separation of the hypocotyls in an upper and lower part showed that the light effect predominantly resulted from augmentation in the upper part, whereas the values remained nearly constant at around 100% of the dark controls for all four STL in lower hypocotyl samples (Supplementary Fig. S4).

### Growth reaction of hypocotyls on external STL application

**Fig. 7:**
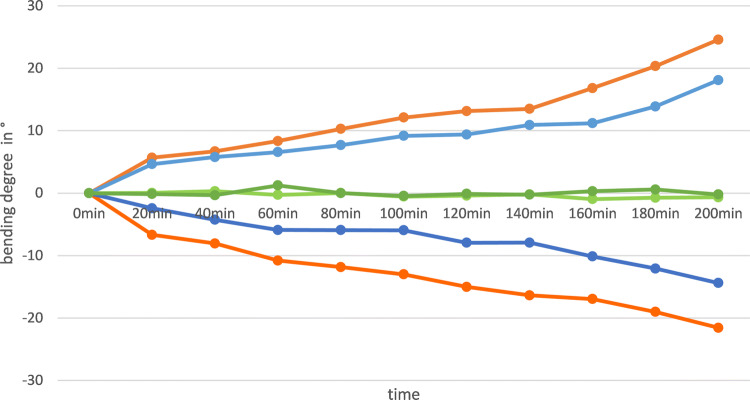
Time course of sunflower hypocotyl bending (deviation in circle from starting position as mean values of *n* = 14 plants per sample and time) after unilateral application of 500 µM costunolide in vaseline (blue lines). Positive values represent left sided, negative values right-sided application. DMSO (orange lines) enforced the effect of costunolide but did not affect the reaction of controls (green lines for samples with and without DMSO, respectively)

When costunolide (500 µM) in a mixture with Vaseline was applied unilateral to the flank of sunflower hypocotyls and the plants were subsequently kept in darkness, a fast bending reaction towards the side of the application (left or right) became visible (Fig. 7). Already 20 min after the treatment, the stems showed a deviation of around 3–5° from the starting position. The angle continuously increased over 200 min to approximately 15°, whereas the hypocotyls of the control plants treated with pure Vaseline kept straight. This effect could be enhanced to nearly 25°, when DMSO (10% final concentration) was added to the vaseline.

## Discussion

### Occurrence of endogenous STL in sunflower

Four studies of the past 20 years reported the presence of endogenous STL in sunflower seedlings (Yokotani-Tomita et al. 1997, 1999; Joel et al. 2011; Raupp and Spring 2013). However, the investigation of their biosynthesis and occurrence in stems, roots and cotyledons of different developmental stages remained fragmentary, on the one hand because of lacking information on the particular enzymes involved in the biosynthesis and on the other hand due to difficulties to identify and quantify the very small amounts of the STL in inner tissues. Joel et al. (2011) showed that dehydrocostuslactone occurred in ng to low µg amounts per g FW in all plant parts of 5–6 days old sunflower seedlings with a decreasing gradient from cotyledons to roots. Our current study now revealed that all four known STL 8-epixanthatin, tomentosin, costunolide and dehydrocostuslactone are present in the different organs of sunflower seedlings. Thereby, their concentration seemed to be dependent on the analyzed tissue, as well as on the developmental stage of the plant. The xanthanolides 8-epixanthatin and tomentosin, for instance, were highly abundant in young cotyledons and stems, but their concentration decreased over time from the 3rd to the 7th day after germination (see Fig. 3). However, the significance of this trend remained uncertain due to the high variation of the measured amounts between different experiments. This also accounted for dehydrocostuslactone, which was found in higher amounts in roots than in hypocotyls or cotyledons. Interestingly, the four compounds were also present in non-germinated seeds where their relatively high concentration (1.5 µg/g FW) dropped by a factor of 3 within the first 24 h of germination. This suggests that the internal STL of the non-germinated seeds may represent a deposit derived from seed ripening in the inflorescence, while the compounds found in 3 days old seedlings may have been predominantly synthesized after germination.

Semi-quantitative expression studies monitoring enzymes of the endogenous STL biosynthesis indicated that the biosynthesis is likely to take place in the same tissues where the compounds occur (Fig. 4). Moreover, differences in the expression between plant tissues of different age supported the hypothesis of developmental regulation in the three plant organs. *HaGAO*, the gene for catalyzing the key step in the pathway of all four compounds, was found highly expressed in cotyledons and roots, with decreasing intensity between the first 5 days of plant development (see Fig. 4). This tendency was confirmed by additional qPCR measurements and correlated positively with the metabolic profiles. The expression of *HaGAO* and *HaCOS* decreased between 48 and 120 h after germination in cotyledons, but seemed to increase again after 8 days (Fig. 5). Only a few studies have been published, trying to correlate the STL metabolic profile with the spatial and developmental expression of genes involved in the STL biosynthesis. Majdi et al. (2014) showed that the expression of the terpene synthase gene *TpGAS* from *Tanacetum parthenium* decreased from young to older leaves. However, the absence of expression in the stem and root suggests that the transcription in leaves did not originate from inner tissues, but from epidermal trichomes, which are well known to be denser and physiologically more active in developing leaves than in fully expanded ones (Aschenbrenner et al. 2015; Bombo et al. 2016; Silva et al. 2017). Testone et al. (2016) showed that *GAS* and *GAO* expression in chicory positively correlated with the STL level in the stem and suggested that MYB and bHLH factors may be involved in the regulation of the pathway. An interesting aspect was introduced by Li et al. (2014) who found that the upregulation of xanthanolide biosynthesis genes in glandular cells of *Xanthium strumarium* was influenced by the hormonal activity of gibberellic acid GA_3_. Whether gibberellic acid (GA) could also play a role in the regulation of STL genes expressed in sunflower seeds and early seedling stages has not been shown yet, but it appears possible in the view that GA is involved in the germination and growth developmental process of sunflower seedlings (Pearce et al. 1991; Bianco et al. 1994). A question remains on the origin and function of the expressed STL genes found in non-germinated seeds. Are they residues of a fading STL biosynthesis during seed maturation (a process that would explain the presence of STL in non-germinated seeds)? Or do they represent a deposit of long-lasting RNA in the embryo to enable faster synthesis at the beginning of germination than by de novo transcription? Many overexpressed RNAs in non-germinated seeds were reported from sunflower and their function, among others, includes secondary metabolism (Xia et al. 2018).

### Influence on plant growth

The idea that an endogenous STL could be involved in auxin-induced growth processes in sunflower stems was published by Shibaoka (1961), who showed that a potential inhibitor was transported from leaves into the stem and that illumination of leaves enhanced the growth inhibition. With the detection of 8-epixanthatin, Yokotani-Tomita et al. (1997) had found a first candidate for such a function. They reported a positive correlation between the STL concentration in the irradiated flank and the phototropic curvature of the stem, and finally could simulated growth inhibition by unilateral external application of 8-epixanthatin (Yokotani-Tomita et al. 1999). Our own results confirmed the fast light-dependent increase of 8-epixanthatin in the hypocotyl and showed a similar effect on tomentosin, the second compound deriving from the xanthanolide pathway via *HaG8H* expression. On the other hand, dehydrocostuslactone and costunolide were almost not affected by the irradiation of the cotyledons with blue light (see Fig. 6). This shows that the germacrolide and the xanthanloide pathways are influenced differently and suggests that 8-epixanthatin and/or tomentosin rather than costunolide or dehydrocostuslactone could be the putative inhibitor predicted by Shibaoka (1961). The question if the xanthanolides were transported from the cotyledons to the hypocotyl or were directly produced there, could not be solved with the applied experimental design. Although their occurrence in root exudates (Raupp and Spring 2013) indicates the general motility of STL in the plant, but since this process most likely involves apoplastic diffusion it appears too slow for the observed fast increase of STL in the hypocotyl after irradiation. The light effect on the STL concentration occurred within the first 120 min after illumination and correlated with the fast reduction of the hypocotyl growth (Fig. S2). This corroborates with the time frame for the increase of 8-epixanthatin in the hypocotyl irradiation shown by Yokotani-Tomita et al. (1999).

The external unilateral application of costunolide (500 µM) on sunflower hypocotyls led to a similar curving of the organ through growth inhibition as had been reported for 8-epixanthatin (200 µM) by Yokotani-Tomita et al. (1999). The relatively high concentrations required for this test can be explained by the diffusion barrier caused by the cuticle. This is supported by the observed effect of DMSO which accelerated and strongly enhanced the curving (Fig. 7). Although not tested here, it can be assumed, that tomentosin and dehydrocostuslactone would lead to similar growth inhibitions. All four endogenous STL possess a α,β-unsaturated-γ-lactone as a functional group which enables SH-alkylation, a feature that had previously been suggested for the growth inhibition of sunflower hypocotyls and oat coleoptiles by STL (Spring and Hager 1982; Spring et al. 1988). As a possible mechanism for this process, the inhibition of the basipetal auxin transport by inhibitors had been supposed (Hager 1971). The ability to inhibit the polar auxin transport has recently been shown for dehydrocostuslactone (isolated from costus root oil) in a radish hypocotyl bioassay (Ueda et al. 2013). This was confirmed by Toda et al. (2019) who studied the growth inhibition of dehydrocostuslactone on etiolated pea seedlings and correlated the effects with the reduction of gene expression for plasma membrane located proteins of the auxin transport. Although not tested yet, it can be assumed that this activity can also be achieved with the three other endogenous STL of sunflower.

### Outlook

Although primarily aimed to assess of the spatial and developmental synthesis of endogenous STL in sunflower, results of the current study together with previously reported data support the assumption of a physiological role of STL in plant growth regulation. This raises questions on possible mechanisms which need to be addressed in the future. The fact that the four STL stimulate germination of sunflower broomrape (Raupp and Spring 2013) in a similar way as GR24, a synthetic strigolactone, favors the suggestion of Joel et al. (2011), that both types of substances may use the same receptor mechanism. Candidates for such a receptor were found in Orobanchaceae as well as in many other plant families. Strigolactone receptors (named KAI2 and D14) from *Arabidopsis thaliana* were characterized as α/β-hydrolases. After binding of suitable ligands, they mediate germination response or controlled shoot branching (Conn et al. 2015). Karrikins (butenolide derivatives, originally found in smoke) are such ligands in the environment which can be taken up by seeds of some plants, but in *A. thaliana,* strigolactones were shown to act similarly as an endogenous signal. Within recent years, an intensive search for additional ligands has been undertaken and the results have broadly been reviewed (Machin et al. 2019; Yang et al. 2019; Yao and Waters 2020). It may be speculated, that STL could play such a role as ligands in sunflower, due to structural similarities with strigolactones such as similar molecular volume, similar polarity (Galindo et al. 2002) and the capability of nucleophilic reaction (Zwanenburg et al. 2009). It will be of interest to perform ligand-receptor studies with STL and to screen other Asteraceae for similar, yet unknown endogenous STL with potential hormonal function. GAO, the key enzyme for STL biosynthesis was shown to be evolutionarily conserved in Asteraceae since the basal tribe of Barnadesoideae (Nguyen et al. 2010), and this will not be without cause.

#### *Author contribution statement*

All authors contributed to the study conception and design. Material preparation, data collection and analysis were performed by KS, NDL, JS, CM, JW, MF and OS. The first draft of the manuscript was written by OS and all authors commented on previous versions of the manuscript. All authors read and approved the final manuscript.

## Electronic supplementary material

Below is the link to the electronic supplementary material.

**Fig. S1** Average length of sunflower hypocotyls of 5 days old plants at the beginning (T_0_) and end (T_48_) of illumination with 14 h per day white light (100 µmol m^−2^ s^−1^; 380-750 nm) compared to dark controls (red line) (EMF 112 kb)

**Fig. S2** Morphological development of sunflower plants. **a** Plants raised for 5 days in darkness and then for 48 h in white light (100 µmol m^−2^ s^−1^; 380-750 nm, 14 h per day). **b** Plants continuously raised in darkness for 7 days (photographed under green light). **c** Lack of chlorophyll and bending of the cotyledon hook in the etiolated dark controls. Bar equals 1 cm (EMF 2875 kb)

**Fig. S3** Schematic illustration of the measurement of hypocotyl bending after unilateral application of costunolide. The deviation was determined every 20 min as an angular degree between the initial hypocotyl position at t_0_ (red line) and the actual position (green line, theoretical straight connection between the lower and upper end of the hypocotyl) (EMF 283 kb)

**Fig. S4** Relative concentration of sesquiterpene lactones in upper and lower parts of the sunflower hypocotyl (hypo) after 2 h and 4 h of illumination. Dark controls are defined as 100%. Mean values ± SE (*n* = 3) are displayed. COS, costunolide; DCL, dehydrocostuslactone; 8-EPI, 8-epixanthatin; TOM, tomentosin (EMF 273 kb)

Supplementary material 5 (DOCX 386 kb)

## Abbreviation

STL: Sesquiterpene lactones
