# Supplementary material for: Spatial and developmental synthesis of endogenous sesquiterpene lactones supports function in growth regulation of sunflower
Source: Planta. 2020 Jun 5;252(1):2. doi: 10.1007/s00425-020-03409-y (PMC7275010; doi:10.1007/s00425-020-03409-y)

**Table S1** Extraction efficacy for STL extraction from cotyledons and hypocotyl/root tissues, respectively. Values are given as mean and SE of *n* = 3 experiments

|  | **Extraction efficacy in %** | | | |
| --- | --- | --- | --- | --- |
| **Extracted tissue** | **8-epixanthatin** | **tomentosin** | **costunolide** | **dehydrocostuslactone** |
| cotyledons | 62 ± 4 % | 75 ± 8 % | 29 ±7 % | 52 ± 6 % |
| hypocotyl or root | 64 ± 5 % | 76 ± 1 % | 20 ± 4 % | 20 ± 3 % |

**Table S2** Primers used for qPCR.

| **Gene**  (Accession-Nr.) | **Primer**  (5´–3´) | **Size**  (bp) | **Ta (^a^)**  (°C) | **E**  (%) | **R^2^** | **Refe-**  **rence** |
| --- | --- | --- | --- | --- | --- | --- |
| HaGAO/  CYP71AV6  (GU256646) | CCGCCCCAAACGGAAGATACTC  GATCCCGAATACTGGAAAGACGC | 85 | 60 | 89.0 | 0.999 | Frey et al. 2018 |
| HaG8H/ CYP71BL1  (HQ439590) | TACGTTAGGCGATGTTAGCGAAGACCACGTGGTCTACTTCTGTGTTCCCTCAA | 214 | 64 | 91.2 | 0.997 | Frey et al. 2018 |
| Actin  (AF282624) | GCCGTGCTTTCTCTTTATGCCAGCGACCAGCGAGATCAAGACGAAG | 137 | 60 | 89.8 | 0.999 | Frey et al. 2018 |
| EF 1α  (AY094064) | ACCAAATCAATGAGCCCAAGAGACCCATACCGGGCTTGATCACACCAG | 131 | 60 | 93.8 | 0.998 | Frey et al. 2018 |
| α-tubulin  (AF401481) | TGCCGTTTCAGAGGTTTTCAGTCGCGCCTTCTTCCATCCCTTCACC | 104 | 60 | 84.8 | 0.985 | Frey et al. 2018 |
| HaCOS/  CYP71BL9  (^b^) | GATACTTATAAATGCGTGGGCTTGTGC GGCCCCAAACGGAAGGAACTC | 136 | 64 | 95.9 | 0.998 | this paper |

(^a^) Ta: annealing temperature, E: amplification efficiency, *EF 1α elongation factor*, *HaGAO* *Helianthus annuus germacrene A oxidase*, *HaG8H Helianthus annuus germacrene A acid 8β-hydroxylase, HaCOS Helianthus annuus costunolide synthase.*

(^b^) GeneBank number not assigned, yet (publication of enzyme in separate manuscript currently under review).

**Table S3** LSD Test for 8-Epixanthatin and Tomentosin values of 2h light versus 2h dark

Signifikanztest 8-EPI: 2h Lichtwert ist nicht signifikant höher als 2h Dunkelwert; p=0,1


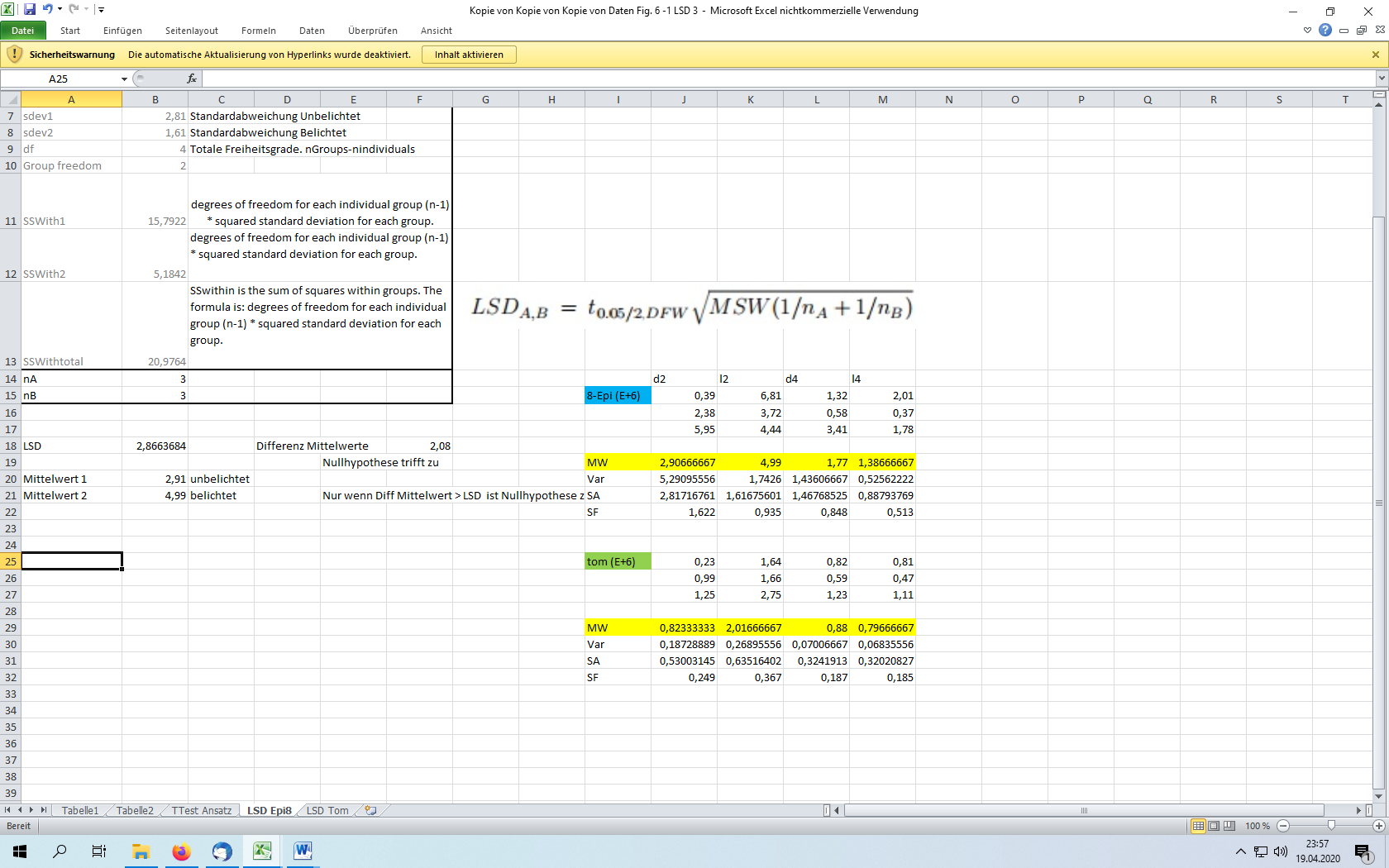


Signifikanztest TOM: 2h Lichtwert ist signifikant höher als 2h Dunkelwert; p=0,05


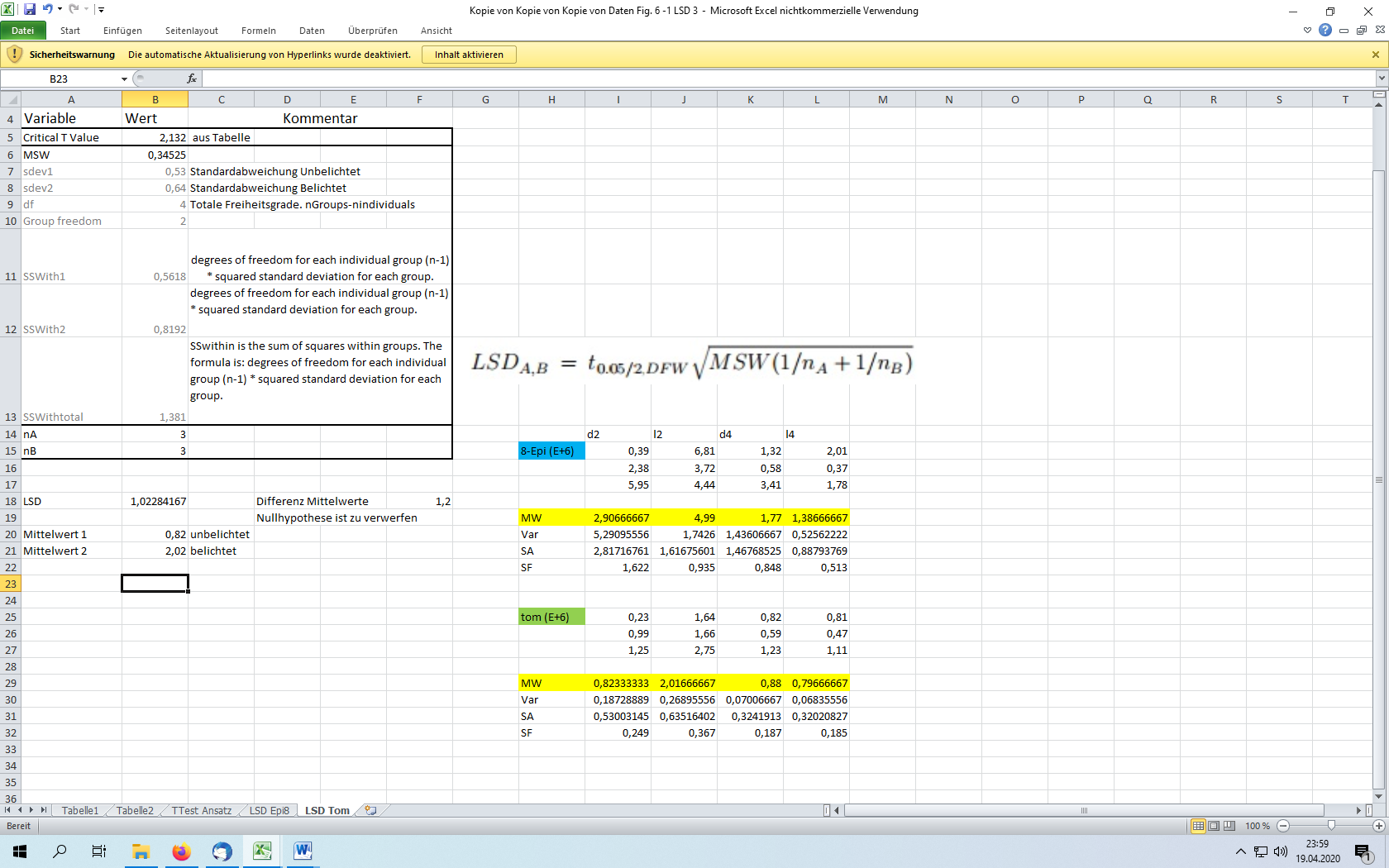

Supplement: Supplementary file 5 — Supplementary material 5 (DOCX 386 kb) [file 425_2020_3409_MOESM5_ESM.docx]
